# Supplementary material for: Engineered Phage Endolysin Eliminates Gardnerella Biofilm without Damaging Beneficial Bacteria in Bacterial Vaginosis Ex Vivo
Source: Pathogens. 2021 Jan 8;10(1):54. doi: 10.3390/pathogens10010054 (PMC7830407; doi:10.3390/pathogens10010054)
Supplement: Supplementary file 1 [file pathogens-10-00054-s002.pdf]

```
CLUSTAL O(1.2.4) multiple sequence alignment

EL11_EPI51560.1          MSKRGIDVSVWQGDIDFNAVKASGVEFVIIRAGYGIGHKDKWFEQNYRKAKTTGLDVGAY 60
EL3_WP_065189629.1       MSKKGIDVSVWQGDIDFNSVKASGVEFVIIRAGYGIGHKDKWFEENYRKAKTAGLDVGSY 60
EL10_WP_064340486.1      MSKRGIDVSVWQGDIDFNAVKASGVEFVIIRAGYGIGHKDKWFEENYRKAKTVGLDVGAY 60
EL12_WP_048730021.1      MSKKGIDVSVWQGDIDFNAVKASGVEFVIIRAGYGIGHKDKWFEENYRKAKTAGLDVGSY 60
EL8_EPI52092.1           MSKKGIDVSEWQGDIDFNAVKASGVEFVIIRAGYGIGCKDKWFEQNYRKAKTAGLDVGAY 60
EL5_EPI54247.1           MSKKGIDVSEWQGDIDFNAVKASGVEFVIIRAGYGIGCKDKWFEQNYRKAKTAGLDVGAY 60
EL7_WP_004104952.1       MSKKGIDVSEWQGDIDFNAVKASGVEFVIIRAGYGIGCKDKWFEQNYRKAKTAGLDVGAY 60
EL4_WP_004111234.1       MSKKGIDVSEWQGDIDFNAVKASGVEFVIIRAGYGIGCKDKWFEQNYRKAKTCGLDVGAY 60
EL6_EIK79883.1           MSKKGIDVSVWQGDIDFNAVKASGVEFVIIRAGYGIGCKDKWFEQNYRKAKTCGLDVGAY 60
EL1_AEF31373.1           MSKKGIDVSEWQGDIDFNAVKASGVEFVIIRAGYGIGCKDKWFEQNYRKAKTAGLDVGAY 60
EL9_WP_004118369.1       MSKKGIDVSEWQGDIDFNAVKASGVEFVIIRAGYGIGCKDKWFEQNYRKAKTCGLDVGAY 60
EL2_WP_065189413.1       MSKRGIDVSEWQGDIDFNAVKASGVEFVIIRAGYGIGCKDKWFEQNYRKAKTCGLDVGAY 60
EL13_WP_076002731.1      MSKKGIDVSVWQGDIDFNAVKASGVEFVIIRAGYGIECKDKWFEQNYRKAKTAGLDVGAY 60
EL14_WP_076002856.1      MSKKGIDVSVWQGDIDFNAVKASGVEFVIIRAGYGIGCKDKWFEQNYRKAKTCGLDVGAY 60
                         ***:***** ********:*****************  ******:******* *****:*

EL11_EPI51560.1          WYSYASSAGEAAEEAQSCVNILSGKSFEYPVYFDLEEKSQLNRGRDFCDSLITSFCNKLE 120
EL3_WP_065189629.1       WYSYASSAGEASEEAQSCVNILSGKSFEYPIYFDLEEKSQLNRGRDFCDSLITSFCNKLE 120
EL10_WP_064340486.1      WYSYASSAGEASEEAQSCVNILSGKSFEYPVYFDLEEKSQLNRGRDFCDSLITSFCNKLE 120
EL12_WP_048730021.1      WYSYASSAGEVALEAQSCVNILSGKSFEYPVYFDLEEKSQLNRGRDFCDSLITSFCNKLE 120
EL8_EPI52092.1           WYSYANSSSEAAEEAQSCVNMLSGKSFEYPVYFDLEEKSQLNRGRAFCDSLITSFCSKLE 120
EL5_EPI54247.1           WYSYANSSSEAAEEAQSCANMLSGKSFEYPVYFDLEEKSQLNRGRAFCDSLITGFCSKLE 120
EL7_WP_004104952.1       WYSYANSASEAAEEAQSCANMLSGKSFEYPVYFDLEEKSQLNRGRAFCDSLITSFCSKLE 120
EL4_WP_004111234.1       WYSYANSGFEAAEEAQSCVNMLSGKSFEYPVYFDLEEKSQLNRGRAFCDSLITSFCSKLE 120
EL6_EIK79883.1           WYSYANSGFEAAEEAQSCVNMLSGKSFEYPVYFDLEEKSQLNRGRAFCDSLITSFCSKLE 120
EL1_AEF31373.1           WYSYANSGFEAAEEAQSLMNMLSGKSFEYPVYFDLEEKSQLNRGRAFCDSLITSFCNKLE 120
EL9_WP_004118369.1       WYSYANSGFEAAEEAQSCVNMLSGKSFEYPVYFDLEEKSQLNRGRVFCDSLITSFCNKLE 120
EL2_WP_065189413.1       WYSYANSGFEAAEEAQSCVNMLSGKSFEYPVYFDLEEKSQLNRGRAFCDSLITGFCNKLE 120
EL13_WP_076002731.1      WYSYANSGFEAAEEAQSCVNMLSGKSFEYPVYFDLEEKSQLNRGRAFCDSLITSFCNKLE 120
EL14_WP_076002856.1      WYSYANSGFEAAEEAQSCVNMLSGKSFEYPVYFDLEEKSQLNRGRAFCDSLITSFCNKLE 120
                         *****.*. *.: ****  *:*********:************** *******.**.***

EL11_EPI51560.1          TYGYYAGFYTSLSVANNLVSSHVRDRYALWIAQWNTHCDYQGSYGLWQYSSSGSVDGIAG 180
EL3_WP_065189629.1       ACGYYAGFYTSLSVANNLVSSHVRDRYALWIAQWNTHCSYQGSYGLWQYSSSGSVNGIAG 180
EL10_WP_064340486.1      ACGYYAGFYTSLSVANNLVSSHVRDRYALWIAQWNTHCSYQGSYGLWQYSSSGSVNGIAG 180
EL12_WP_048730021.1      ACGYYAGFYTSLSVANNLVSSHVRDRYALWIAQWNTHCSYQGSYGLWQYSSSGSVNGIAG 180
EL8_EPI52092.1           TYGYYAGFYTSLSTANNLVSSHVRNRYALWIAQWNTHCSYQGSYGLWQYSSNGSVPGVAG 180
EL5_EPI54247.1           ACGYYAGFYTSLSTANNLVSAHVRNRYALWIAQWNTHCSYQGSYGLWQYSSNGSVPGVAG 180
EL7_WP_004104952.1       TYGYYAGFYTSLSTANNLVSSHVRNRYALWIAQWNTHCSYQGSYGLWQYSSSGSVPGVAG 180
EL4_WP_004111234.1       TYGYYAGFYTSLSVVNNLVSAHVRDRYALWIAQWNTHCSYQGSYGLWQYSSSGSVPGVAG 180
EL6_EIK79883.1           SCGYYAGFYTSLSTANNLVSAHVRNRYALWIAQWNTHCDYQGSYGLWQYSSSGSVPGVAG 180
EL1_AEF31373.1           ACGYYAGFYTSLSTANNLVSAHVRNRYALWIAQWNTHCNYQGSYGLWQYSSNGSVPGVAG 180
EL9_WP_004118369.1       ACGYYAGFYTSLSTANNLVSSHVRNRYALWIAQWNTHCDYQGSYGLWQYSSSGSVPGVAG 180
EL2_WP_065189413.1       SCGYYAGFYTSLSTANNLVSAHVRNRYALWIAQWNTHCSYQGSYGLWQYSSSGSVPGVAG 180
EL13_WP_076002731.1      SCGYYAGFYTSLSTANNLVPAHVRNRYALWIAQWNTHCDYQGSYGLWQYSSSGSVPGVAG 180
EL14_WP_076002856.1      ACGYYAGFYTSLSTANNLVSAHVRNRYALWIAQWNTHCSYQGSYGLWQYSSSGSVPGVAG 180
                         : ***********..**** :***:*************.************.*** *:**

EL11_EPI51560.1          RVDMDYTYVDYPSVIKKAGLNGYKNGGSYTAPQTSSIDEVAREVINGDWGNGNERKNRLT 240
EL3_WP_065189629.1       RVDMDYAYVDYPSVIKNAGLNGYQNGGSYTAPQTSSIDEVAREVINGDWGNGNDRKNRLI 240
EL10_WP_064340486.1      RVDMDYAYVDYPSVIKNAGLNGYKNGGSYTAPQISSIDEVAREVINGDWGNGNERKQRLT 240
EL12_WP_048730021.1      RVDMDYAYVDYPSVIKNAGLNGYQNGGSYTAPQTSSIDEVAREVINGDWGNGIERKNRLT 240
EL8_EPI52092.1           RVDMDYAYVDYPSIIKNAGLNGYKNGGSYTAPQTSSIDDVAREVINGAWGNGNERKQRLT 240
EL5_EPI54247.1           RVDMDYAYKDYPSIIKNAGLNGCKNGGSDQAARTSSIDEVAREVINGAWGNGNERKQRLT 240
EL7_WP_004104952.1       RVDMDYAYKDYPSIIKNAGLNGCKNGGSDQAARTSSIDEVAREVINGAWGNGNERKQRLT 240
EL4_WP_004111234.1       RVDMDYAYVDYPSIIKNVGLNGCKNGGSDQATRTSSIDEVAREVINGAWGNGNERKQRLT 240
EL6_EIK79883.1           RVDMDYAYKNYPSIIKNAGLNGCKNGGSNQAARTSSIDDVAREVINGAWGNGNERKQRLT 240
EL1_AEF31373.1           RVDMDYAYVDYPSIIKNAGLNGCKNGGSDQAARTSSIDEVAREVINGAWGNGSTRKQRLT 240
EL9_WP_004118369.1       RVDMDYAYVDYPSIIKNAGLNGCKNGGSDQAARTSSIDEVAREVINGAWGNGSTRKQRLT 240
EL2_WP_065189413.1       RVDMDYAYVDYPSIIKNVGLNGCKNGGSDQAARTSSIDEVAREVINGAWGNGNERKQRLT 240
EL13_WP_076002731.1      RVDMDYAYVDYPSIIKNAGLNGYKNGESHQATRTTSIDEVAREVINGAWGNGNERKQRLT 240
EL14_WP_076002856.1      RVDMDYAYVDYPSIIKNAGLNGYKNGESHQATRTTSIDEVAREVINGAWGNGNERKQRLT 240
                         ******:* :***:**:.**** :** *  * : :***:******** ****  **:**
```

```
EL11_EPI51560.1          SAGYDYTSVQNKVNELLGVKAYRKSVDELAREVIRGTWGNGSTRKQRLTQAGYDYDAVQK 300
EL3_WP_065189629.1       SAGYDYASVQNKVNELLGVKAYRKSVDELAREVIRGTWGNGSMRKHRLTQAGYDYDAVQK 300
EL10_WP_064340486.1      SAGYDYASVQNKVNELLGVKAYRKSVDELAREVIRGTWGNGSTRKQRLTQAGYDYNAVQK 300
EL12_WP_048730021.1      SAGYDYTSVQNKVNELLGVKAYRKSVDELAREVIRGTWGNGKTRKQRLTQAGYDYNAVQK 300
EL8_EPI52092.1           QAGYDYTSVQNKVNKLLGVKACRKSVDELAREVIRGTWGNGNERKNRLTQAGYDYDTVQK 300
EL5_EPI54247.1           QAGYDYTSVQNKVNKLLGVKACRKSVDELAREVIRGTWGNGNERKNRLTQAGYDYDTVQK 300
EL7_WP_004104952.1       QAGYDYTSVQNKVNKLLGVKACRKSVDELAREVIRGTWGNGNERKNRLTQAGYDYDTVQK 300
EL4_WP_004111234.1       SAGYDYASVQNKVNKLLGVKAYRKSVDELAREVIRGTWGNGNERKQRLAQAGYDYDTVQK 300
EL6_EIK79883.1           QAGYDYASVAK------------------------------------------------- 251
EL1_AEF31373.1           SAGYDYASVQNKVNELLGVKACRKSVDELAREVIRGAWGNGSTRKQRLAQAGYDYDTVQK 300
EL9_WP_004118369.1       SAGYDYASVAK------------------------------------------------- 251
EL2_WP_065189413.1       SAGYDYASVQNKVNELLGVKACRKSVDEIAREVIRGTWGNGSTRKQRLTQAGYDYDTVQK 300
EL13_WP_076002731.1      QAGYDYASVQNKVNELLGVKACRKSVDELAREVIRGTWGNGNERKNRLTSAGYDYDTVQK 300
EL14_WP_076002856.1      SAGYDYASVQNKVNELLGVKACRKSVDELAREVIRGAWGNGSTRKQRLTSAGYDYDTVQK 300
                         .*****:** :

EL11_EPI51560.1          RVNELL       306
EL3_WP_065189629.1       RVNELL       306
EL10_WP_064340486.1      RVNELL       306
EL12_WP_048730021.1      RVNELL       306
EL8_EPI52092.1           RVNELL       306
EL5_EPI54247.1           RVNELL       306
EL7_WP_004104952.1       RVNELL       306
EL4_WP_004111234.1       RVNELL       306
EL6_EIK79883.1           ------       251
EL1_AEF31373.1           RVNELL       306
EL9_WP_004118369.1       ------       251
EL2_WP_065189413.1       RVNELL       306
EL13_WP_076002731.1      RVNELL       306
EL14_WP_076002856.1      RVNELL       306
```

Supplementary Figure 1: Wild-type endolysins (EL1-EL14) encoded on prophage-like regions of *Gardnerella* genomes. A multiple sequence alignment was performed by Clustal Omega analysis (https://www.ebi.ac.uk/Tools/msa/clustalo/). The protein accession numbers are given after each endolysin name (e.g. EL1_AEF31373.1). The yellow background indicates the EAD (H-domain), the linker sequence is highlighted in grey, and the cell wall binding domain (B-domain) in blue. The domain regions were predicted by Interpro (https://www.ebi.ac.uk/interpro/).

a **b**

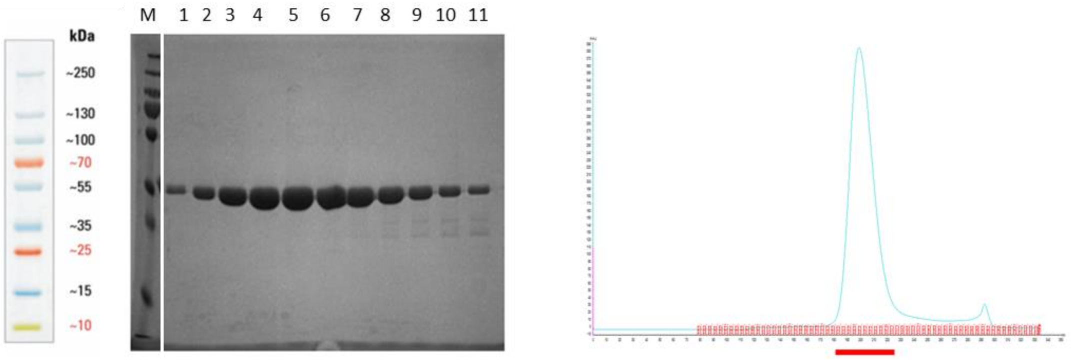

Supplementary Figure 2. (a) PM-477 endolysin protein was recombinantly expressed and purified via Ni-NTA chromatography (FPLC). Fractions obtained from the final size exclusion chromatography (FPLC) were analyzed for purity by SDS-PAGE and Coomassie blue staining. MWM was used to confirm the expected protein size (left). Fractions 1-11 were pooled and used for further experiments. (b) FPLC chromatograph of PM-477 purification on a Superdex 75 column resulting in a defined peak from 182 mL to 222 mL. Red bar represents the fractions 1-11 that were pooled and concentrated to a final volume with a concentration of 0.616 mg/mL (determined by A280 measurement).
